# “Complex abdominal wall” management: evidence-based guidelines of the Italian Consensus Conference

**DOI:** 10.1007/s13304-018-0577-6

**Published:** 2018-09-25

**Authors:** Micaela Piccoli, Ferdinando Agresta, Grazia Maria Attinà, Dalia Amabile, Domenico Marchi, “Complex abdominal wall study” Italian Collaborative Group, Carlo Bergamini, Rossana Berta, Luigi Boccia, Diego Cuccurullo, Valentino Fiscon, Francesco Gossetti, Pierluigi Ipponi, Giovanna Riccipetitoni, Massimiliano Rimini, Domenico Russello, Vincenzo Trapani, Fausto Tricarico, Gregorio Tugnoli, Gianluigi Melotti, Luca Ansaloni, Simona Barlera, Davide Beghelli, Giampiero Campanelli, Fabio Cesare Campanile, Michele Carlucci, Salvatore Casarano, Osvaldo Chiara, Piero Chirletti, Francesco Corcione, Feliciano Crovella, Marina Davoli, Ezio Gianetta, Stefano Giordani, Rita Hervatin, Mauro Longoni, Pietro Maida, Pierluigi Marini, Gabriele Munegato, Paolo Negro, Paolo Pelosi, Diego Piazza, Luigi Presenti, Roberto Rea, Massimo Sartelli, Mario Testini, Andrea Valeri, Nereo Vettoretto, Rodolfo Vincenti

**Affiliations:** 1 Department of General Surgery, General Surgery Unit, New Sant’Agostino Hospital, Via Pietro Giardini, 1355, 41126 Modena, Italy; 2 Department of General Surgery, ULSS19 Veneto, Piazzale degli Etruschi 9, 45011 Adria, Italy; 3 Department of General Surgery, General Surgery Unit, S. Camillo-Forlanini Hospital, Circonvallazione Gianicolense, 87, 00152 Rome, Italy; 4 Department of General Surgery, General Surgery 1, Saint Chiara Hospital, Largo Medaglie D’oro, 9, 38122 Trento, Italy

**Keywords:** Complex abdominal wall, Emergency and elective treatment, Meshes, Laparostomy

## Abstract

To date, there is no shared consensus on a definition of a complex abdominal wall in elective surgery and in the emergency, on indications, technical details, complications, and follow-up. The purpose of the conference was to lay the foundations for a homogeneous approach to the complex abdominal wall with the primary intent being to attain the following objectives: (1) to develop evidence-based recommendations to define “complex abdominal wall”; (2) indications in emergency and in elective cases; (3) management of “complex abdominal wall”; (4) techniques for temporary abdominal closure. The decompressive laparostomy should be considered in a case of abdominal compartment syndrome in patients with critical conditions or after the failure of a medical treatment or less invasive methods. In the second one, beyond different mechanism, patients with surgical emergency diseases might reach the same pathophysiological end point of trauma patients where a preventive “open abdomen” might be indicated (a temporary abdominal closure: in the case of a non-infected field, the Wittmann patch and the NPWT had the best outcome followed by meshes; in the case of an infected field, NPWT techniques seem to be the preferred). The second priority is to create optimal both general as local conditions for healing: the right antimicrobial management, feeding—preferably by the enteral route—and managing correctly the open abdomen wall. The use of a mesh appears to be—if and when possible—the gold standard. There is a lot of enthusiasm about biological meshes. But the actual evidence supports their use only in contaminated or potentially contaminated fields but above all, to reduce the higher rate of recurrences, the wall anatomy and function should be restored in the midline, with or without component separation technique. On the other site has not to be neglected that the use of monofilament and macroporous non-absorbable meshes, in extraperitoneal position, in the setting of the complex abdomen with contamination, seems to have a cost effective role too. The idea of this consensus conference was mainly to try to bring order in the so copious, but not always so “evident” literature utilizing and exchanging the expertise of different specialists.

## Introduction

The management of complex abdominal problems with the “open abdomen” (OA) technique has become a routine procedure in surgery. The number of cases treated with an OA has increased dramatically because of the popularization of damage control for life-threatening conditions, recognition, and treatment of intra-abdominal hypertension and abdominal compartment syndrome and new evidence regarding the management of severe intra-abdominal sepsis [1].

Although OA has saved numerous lives and has addressed many problems related to the primary pathology, this technique is also associated with serious complications. New knowledge about the pathophysiology of the OA and the development of new technologies for temporary abdominal wall closure has helped improve the management and outcomes of these patients [2].

To date, there is no shared consensus on a definition of a complex abdominal wall in elective surgery and in the emergency, on indications, technical details, complications, and follow-up [3]. These thoughts and the desire to establish recommendations based on clinical evidence led to the organization of the Italian Consensus Conference, held in Genoa (Italy) on 24 and 25 June 2015.

The purpose of the Conference was to lay the foundations for a homogeneous approach to the complex abdominal wall with the primary intent being to attain the following objectives:

1. To develop evidence-based recommendations to define “complex abdominal wall”;
2. Indications for open abdomen in emergency and in elective cases;
3. Management of “complex abdominal wall”;
4. Surgical details and indication for use techniques for temporary abdominal closure;
5. Use of biological and synthetic meshes and follow-up.

## Materials and methods

The following parties were involved in the promotion, organization and management of the Consensus Conference (CC): a Promoting Committee (PC), which is composed of several Italian Surgical Societies, a Scientific Committee (SC), which designated the team of Experts (ES), and the Members of the Jury Panel (JP), which formulated the questions.

### Organization of the Consensus Conference

A pre-consensus meeting was held in Rome, on 2 December 2014, in which the SC, the ES, and JP met to properly plan the Conference. The organization of the Consensus and the roles of all involved parties were presented during this meeting.

Issues were exposed and discussed with the ES and JP, and the preparatory timing for the delivery of materials by the ES was defined.

All of the ES’s reports were submitted to the Scientific Secretary by 8 March 2015. The Secretary sent the reports (texts and slides) to all members of the Jury prior to the CC. In the same cases, the ES requested changes and clarifications, which were promptly carried out.

### Holding of the Consensus Conference

The Consensus Conference was held in Genoa on 24–25 June 2015. The ES briefly summarized their reports in a plenary session with the Scientific Committee, the JP, and the audience, and discussion was set up.

### Closing session of the Consensus Conference

At the end of the 1st day of the CC, the JP met to draft the preliminary document. Each chapter was discussed and analyzed, producing a key statement with a grade of recommendations (weak and strong) followed by a commentary to explain the rationale and the level of evidence behind the statement. All key statements were formulated according to a 10% consensus obtained within the whole group. The following day these statements were presented to the audience. Comments from the audience were collected and included in the manuscript wherever they were deemed to be relevant. A final version of the guidelines was prepared a month after the CC and sent to all members of the JP for their final approval. The format of the Consensus Conference was freely adapted from the standards of the National Institute of Health (NIH) and the Italian Health Institute.

### Literature searches and appraisal

The literature research was done by the SC and approved by all the members of the Consensus. The Oxford 2011 for grading clinical studies according to levels of evidence (EL) was used. The primary objective of the search was to identify all clinical relevant randomized controlled trials (RCT). However, reviews, reports, population-based outcomes studies, case series, and case reports were also included. Studies containing severe methodological flaws were highlighted and downgraded as necessary. A systematic review based on a comprehensive literature research up to June 2015 was made on Pubmed and Cochrane Library. We considered both English and Italian language.

We have used as keywords MeSH for example: “abdominal surgery”, “postoperative complications”, “recurrence”, “surgical wall dehiscence”, “open abdomen”, “management, laparostomy”, “damage control surgery”, “ventral hernia AND incisional hernia”, “mesh, biologic mesh, contaminated field, cross-linked, non-cross-linked”, “congenital abdominal wall defect AND biological prosthesis”; “abdominal wall closure AND pediatric transplant”.

## Results

The Jury undertook every reasonable endeavor to answer the following questions:

1A. Definition of “Complex abdomen” in the emergency:

An abdomen may be defined as complex when there is a high-risk of (1) compartment syndrome, (2) suture dehiscence, (3) early re-do laparotomy/second look (STRONG RECOMMENDATION).

These clinical situations are related to patient’s risk factors, disease risk factors and type of surgery. Abdominal wall closure represents a serious problem to solve by the surgeon in a complex surgical situation such as laparotomies in critical patients and in emergency surgery for difficult trauma, large ventral hernia, peritonitis or bowel occlusion. One of the main questions is either if to close the muscular fascia or not and which type of surgical closure is better to minimize the postoperative complications. Despite this problem’s frequency there’s still not a general consensus regarding the criteria to defined “complex” an abdominal wall closure [4, 5] (EL 3).

Several different multifactor scoring systems predictive of abdominal wall suture complications have been proposed in literature such as the VAMC score and the Rotterdam score [5] (EL 3). According to them age > 70 years old, obesity, cigarettes, steroid use or cytostatic, diabetes, malnutrition, ASA III and IV, vascular disease, constipation, ascites, BPCO, hospital length of stay, sepsis and previous laparotomies [4–6] (EL 5) were indicated as patients risk factors. Whereas disease and surgical risk factor connected with abdominal wall suture complications included abdominal trauma, ruptured abdominal aortic aneurysm, retroperitoneal hemorrhage, pancreatitis, peritonitis, bowel occlusion surgery with bowel resection or suture, wound infection, presence of enterocutaneous fistula, large sized abdominal wall > 10 cm in width, multiple ventral defects, loss of domain > 20%, synthetic mesh infection, necrotizing fasciitis, surgical procedure with wound class III (contaminated) or IV (dirty), closure of a laparostomy, head trauma with head hypertension, surgical abdominal closure with component separation or use of synthetic or biological mesh [1, 4–9] (EL 5).

1B. Definition of “Complex abdomen” in elective surgery.

When there are one or more of these conditions: (1) loss of domain > 20%; (2) surgical wound class III (Contaminated) or IV (Dirty) infection; (3) wound ulcers/non-healing wound; (4) closure after open abdomen; (5) the presence of entero-cutaneous fistula; (6) the presence of a previous mesh infection; (7) a recurrent hernia after many previous mesh repair attempts; (8) full-thickness abdominal wall defects with loss of substance; (9) need of intra-peritoneal mesh removal with a wide adhesiolysis (especially when determining enterotomy), an abdomen may be defined as complex in elective surgery (STRONG RECOMMENDATION).

The term ‘‘complex (abdominal wall) hernia” is often used by general surgeons to describe hernias that are technically challenging and time-consuming. Unfortunately, there is not a precise consensus on which should be the criteria that define the complexity. Moreover, in some cases, the complexity is understood only intra-operatively (e.g., bowel perforation, e.g., non-predicted adhesions, etc.) [4] (EL 3).

Four categories are the most frequently discussed in the literature for the definition of ‘‘complex abdominal wall hernia’’.

*Defect size, feature, and location*

An increased hernia size is considered a risk factor for both 30-day readmission due to complications and recurrence [10, 11] (EL 4). The width seems to be more informative than the length of the defect. 10 cm is a useful cut-off point for a complex hernia provided there are other complicating factors [12] (EL 5).

Lumbar, lateral, and subcostal locations of hernias are considered to be complex because of mesh anchorage difficulty and risk of recurrence. Additionally, parastomal hernias might be considered as complex [13] (EL 4).

When a large proportion of the abdominal contents resides permanently in the hernia sac (> 20%), this may be considered as a complex hernia [14] (EL 3). Therefore, a CT scan is mandatory in determining the complexity abdominal wall hernia.

*Contamination and soft tissue condition*

Contamination and subsequent infection are well known to be an important cause of wound dehiscence and re-herniation by disturbing wound healing dynamics [15, 16].

Moreover, as shown in a recent systematic review of PubMed, EMBASE and Cochrane databases evaluating postoperative outcomes reported in patients undergoing contaminated/infected ventral incisional herniorrhaphy [17] (EL 1) {hernias with wound classes III (contaminated) and IV (dirty/infected) has to be considered complex.

Furthermore, the condition of the soft tissue (such as significant loss of skin or presence of a laparostomy covered with a skin graft, ‘battle- scarred’ abdomen, and loss of myofascial tissue due to trauma, tumor resection or debridement) has important consequences for the complexity of surgical treatment.

*Patient history and risk factors*

A recurrent hernia is considered a risk factor for a new recurrence. It is proposed that these patients suffer an underlying dysfunction in the wound healing process, leading to weakened scar tissue [18] (EL 3). Various other patient-related variables are included in several classification systems indicating the following risk factors: age, male gender, chronic pulmonary disease, coughing, ascites, jaundice, anemia, emergency surgery, wound infection, obesity, steroid use, hypoalbuminemia, hypertension, perioperative shock, and type of surgery [5, 19, 20] (EL 3).

*Clinical scenario*

Both an emergency hernia repair including bowel resection and the presence of a previously placed mesh which needs to be explanted for any reason were considered by various groups to be a complex situation [21, 22] (EL 5) due to the necessity, in the latter case, of extensive adhesiolysis, longer operating time and increased risk of inadvertent enterotomies resulting in dense adhesions, fistulas, and infection [23] (EL 2). Besides the clinical scenarios described in the classification systems, also the following complicating situations that necessitate significant pre- and perioperative measures and planning should be added: abdominal wall with multiple hernia defects (e.g., a ‘‘battle-scarred abdomen’’), a non-healing wound, and when no primary closure is possible [24] (EL 3). In particular, the presence of entero-cutaneous fistulas is known to coincide with high morbidity, mortality and fistula and hernia recurrence rates [25] (EL 4).

2. Indications to open abdomen in emergency and trauma.

In conditions of Damage Control Surgery, the preventive Open Abdomen is indicated in the presence of packing that should be removed, massive hemorrhage, severe peritonitis, major abdominal and retroperitoneal tissue edema, loss or altered tropism of fascia or when it is necessary a second look (STRONG RECOMMENDATION).

The therapeutic Open Abdomen both in emergency surgery and in trauma is indicated in cases of compartment syndrome in which medical treatment failed (STRONG RECOMMENDATION).

Although the therapeutic Open Abdomen is also recommended the cases of intracranial hypertension not responsive to medical therapy: cause the paucity of cases, it is not possible to give a greater level of evidence (WEAK RECOMMENDATION).

*Are the indications to laparostomy the same in emergency surgery and trauma surgery?*

Although the mechanism is different, some patients with nontraumatic, but surgical emergency diseases reach the same pathophysiological end-point of trauma patients who develop a coagulopathy not mechanical and/or severe state of sepsis [26, 27]. For this reason, the indications for use of the laparostomy both preventive or decompressive of the abdominal cavity are the same both in emergency and trauma surgery even if with the lower level of evidence [27, 28] (EL 3).

*When is a preventive laparostomy indicated?*

The literature does not clarify definitively the real benefits of preventive laparostomy in patients with high-risk. Furthermore, for what concerns the traumatic pathology (and others) treated according to the principles of Damage Control Surgery, this should not be considered strictly as an indication, but as an integral part of the technique.

The preventive laparostomy is indicated in case of Damage Control Surgery [28–32] (EL 2) and in severe abdominal hypertension [28–30, 33, 34] (EL 3): in the first case, the laparostomy is an integral part of the surgical technique in the context of a planned re-operation, in the second one the positive effects of abdominal decompression on hemodynamics and on respiratory and renal functions are obvious, but there is no evidence of what is the maximum value of intra-abdominal pressure which can be tolerated without risk at the end of the first operation; furthermore it is not known the cut-off value that makes absolutely indicated a preventive laparostomy.

The preventive laparostomy is also indicated after a trauma that results in greater tissue loss or after surgery with precarious band conditions. A preventive laparostomy seems indicated even in the presence of visceral perforation with extensive contamination of the peritoneal cavity or intense edema of the bowels and of the retroperitoneum [27, 31–33, 35–37] (EL 2).

As an outcome, the high morbidity (and mortality) in patients undergoing a preventive laparostomy derived from the critical condition of the patients rather than from specific risk factors of the technique.

*When is a decompressive laparostomy indicated?*

Decompressive laparostomy should be considered in cases of abdominal compartment syndrome in adult patients with critical conditions.

In the case of medical treatment or less invasive methods failure, such as percutaneous drainage of abdominal abscess, surgical decompression is needed; however, the improvement of the organ functions are not strictly related to the decline of the intra-abdominal pressure [28–30, 33, 34, 38] (EL 2).

Severe intracranial hypertension is a further indication to decompressive laparostomy as a help for the medical treatment [39] (EL 3).

3. Temporary Abdominal Closure (TAC) technique.

In the absence of sepsis, the Wittmann patch and Negative Pressure Wound Therapy (NPWT) abdominal dressing offered the best outcomes. In the presence of sepsis, NPWT had the highest delayed primary closure rate and lowest mortality, especially when associated with continuous fascial retraction to achieve delayed fascial closure and a reduction of the risk of enteroatmospheric fistula (WEAK RECOMMENDATION).

Protected non-absorbable and absorbable meshes can be used for temporary abdominal closure. Absorbable meshes may be left in place at the closure of abdomen, whereas non-absorbable materials usually need to be removed (WEAK RECOMMENDATION).

All TAC systems that do not prevent retraction of the fascia should be used if a definitive closure is possible in a short time. They are simple, inexpensive and prevent bowel desiccation allowing the conservation of electrolytes and thermal effects (STRONG RECOMMENDATION).

The main indications of temporary abdominal closure (TAC) technique are prophylactic abdominal decompression, planned repeated explorations of the peritoneal cavity and the treatment of the abdominal compartment syndrome. Re-exploration of the abdomen generally occurs after 24–72 h [8, 40, 41] (EL 3). A definitive closure can be obtained only when the abdominal acute condition and bowel edema are resolved and the intra-abdominal pressure remains under 25–30 mmHg [8, 41, 42] (EL 4). Definitive abdominal reconstruction is the ultimate goal but we must consider another endpoint in evaluating the TAC techniques, such as fistula and abscess rate and associated survival. Any technique has different features and should be chosen according to the specific circumstance [41].

*What kind of technique is preferred in the field of not infected abdomen?*

The Wittmann patch and the negative pressure wound therapy (NPWT) had the best outcome followed by meshes. Wittmann Patch can be used only as a temporary TAC system to prevent lateral fascial retraction and permits adjustments according to the intra-abdominal pressure. It facilitates re-operation, but it does not allow effective drainage of intra-abdominal infectious fluid. Furthermore, it is related to an increased rate of enterocutaneous fistula. In addition, there is a concern that the sutures on the fascia might cause ischemic damage to the edges, making the definitive closure more difficult [8, 30, 42–45] (EL 3).

*What kind of technique is preferred in the field of the infected abdomen?*

In the case of the infected/contaminated abdomen, NPWT techniques play an important role: removal of peritoneal fluid, inflammatory mediators reduces the concentration of cytokines in the bloodstream and approximation of the fascial edges [46] (EL 1) [47] (EL 3). There are different types of NPWT techniques: Barker, Vacuum Assisted Closure (VAC) abdominal dressing system and ABThera system. ABThera seems to achieve a better performance concerning: survival, the rate of enterocutaneous fistulas and removing of peritoneal fluids [48] (EL 4–5). Furthermore, ABThera system, compared to the others, demonstrated to produce a smaller negative pressure on the bowel loops, with a lower enterocutaneous fistula rate fluids [48] (EL 5). Attention has to be paid to incomplete hemostasis and high-risk anastomosis fluids [48] (EL 5). Dressing changes are performed every 1–3 days or even more frequently when there is an abdominal contamination or infection. Moreover, the combination of ABThera and abdominal re-approximation anchor system (ABRA) has been demonstrated to be ideal in managing patients who may not achieve primary fascial closure with ABThera alone. In fact, ABRA helps to overcome ABThera’s system limited ability to stabilize the fascia in some patients [8, 45, 48–52] (EL 4–5).

*When can be used a technique that does not prevent fascial retraction?*

Bogota bag, steridrape, sylastic sheet, silicon sheeting, skin approximation, and zipper might be valuable in cases with damage control for intra-abdominal bleeding in which definitive closure is anticipated within the next 2 or 3 days [9, 53, 54] (EL 3). No fluid removal is possible and should not be left in place more than 3–14 days [1, 53, 54] (EL 3).

*When can be used meshes in TAC?*

Absorbable meshes are always related to the development of a large ventral hernia, needing an additional operation. Their function is to prevent fascial retraction while facilitating re-operation and a stepwise approximation of the fascial edges. It does not allow peritoneal fluids remove so it should not be used in case of sepsis. Meshes combined with negative pressure therapy (NPT) technique, in some studies, have been reported to improve the primary fascial closure rates. They carry a high incidence of enterocutaneous fistula. In a case of contamination, it should be used a biologic mesh as a temporary or definitive solution. Despite their cost and low availability, biologic material seems to perform better in a condition of infection as it clear bacteria and revascularized. Further studies should be done to assess the role of biologic meshes in TAC [29, 55–58] (EL 4–5).

4. Decision-making in the management of open abdomen.

It’s recommended to close the abdomen as quickly as possible. It is suggested within 9 days (STRONG RECOMMENDATION).

It’s recommended to revise TAC system every 24–72 h and to explore the abdomen only if it’s necessary. A restrictive fluid resuscitation and a strict control of the infections is suggested (WEAK RECOMMENDATION).

Trauma surgeons have gained a huge amount of experience with multiple techniques used to achieve abdominal closure of the open abdomen, but questions still remain: How long can the abdomen remain open? When does the risk of complications begin to increase? Is there a specific technique that is better than the rest for closing the open abdomen? At what point should all attempts at delayed fascial closure be abandoned and a planned ventral hernia performed [59]?

The optimal timing for abdominal closure has not been determined and varies widely between different series. Miller et al. demonstrated that DAFC (delayed abdominal closure) before 8 days was associated with fewer complications: 12% in those closed before 8 days and 52% in that closed after 8 days. Lambertz et al. showed a similar result and underlined that the presence of peritonitis is not a negative prognostic marker concerning fascial closure, while the presence of pancreatitis seems to influence the rates of fascial closure negatively [60–64] (EL 4).

In 2009, the Open Abdomen Advisory Panel [65] was formed to identify the core principles of open abdomen management. They emphasized that throughout the management of a patient with an open abdomen, “a central goal is the closure of the abdominal defect as quickly as possible without precipitating abdominal compartment syndrome”, defining it as the most effective way to reduce the complications associated with the OA. A combination of three strategies such as (1) avoidance of excessive fluid resuscitation, (2) use of effective NPT dressings for temporary abdominal wall closure and (3) use of biological materials in appropriate cases for definitive fascial closure [66] (EL 4) has been indicated to achieve this purpose.

The timing of the re-exploration of an abdomen generally occurs after 24–72 h. At this time the initial adhesions between the viscera and the peritoneal surface are easily separable and the access to any space with no-traumatic dissection is quite feasible. In this stage, it is possible to perform total exploration of the abdominal cavity. After, the intra-abdominal structures begin to adhere to each other, cemented by massive adhesions, vascularized and difficult to separate (frozen abdomen). A definitive closure can be obtained only when the abdominal pathology and bowel edema are resolved and the intrabdominal pressure remains under 25–30 mmHg [8, 41] (EL 4).

Greater number of serial abdominal explorations, the fluid volume overload, abdominal abscess and enterocutaneous fistulae were negatively associated with primary fascial closure [67] (EL 4).

In the setting of ongoing intra-abdominal infection or the formation of an enterocutaneous fistula abdominal fascial closure is often not possible. The fascial closure may not be possible because of ongoing visceral edema with loss of abdominal domain or from loss of fascia from infection. At this point, a fascial bridge closure of the resulting abdominal fascial defect may be considered. The surgeon is limited in the available surgical options: (1) bridge repair of the fascial defect using a mesh to create a bridge closure, (2) performing an acute abdominal wall reconstruction using most commonly a version of component separation, or (3) a planned ventral hernia using adsorbable mesh (vicryl or dexon mesh and skin graft or skin flaps) [59] (EL 4–3).

5–6. When a biologic graft may or must be used in a complex abdominal wall repair?

The biological prosthesis should be implanted only in clinical cases where is present a contaminated or potentially contaminated surgical field (STRONG RECOMMENDATION).

The use of not cross-linked material should be associated with abdominal wall midline restoration, with or without component separation, due to a higher recurrence rate in the bridge repair without midline restoration (WEAK RECOMMENDATION).

The cross-linked material could be used in case of midline restoration. It should be used in case of bridging thanks to a higher tensile strength (WEAK RECOMMENDATION).

Waiting for stronger scientific evidence, the surgeon should orientate their choice case by case, evaluating the main target: resistance to tissues tension or prevention of mesh infections.

Despite advances in surgical technique and prosthetic technologies, repair of complex anterior abdominal wall defects, particularly when bacterial contamination is present or the risk of infection is high, remains a complex and challenging surgical undertaking. Although permanent prosthetic mesh is considered the gold standard for minimizing hernia recurrence, their non-absorbable characteristics may cause potential problems, resulting in erosion into the abdominal viscera, bowel fistulae, and chronic pain, which can lead to more complex and costly surgery. Moreover, placement of synthetic mesh is sometimes imprudent, especially in high-risk contaminated wounds. Consequently, within the past decade, several bioprosthetic materials have been developed to support tissue reconstruction while minimizing the potential complications that come from foreign material reactions of synthetic mesh and their potential to act as a site for infection. As all mesh prostheses, the purpose of the biologic graft is to assist a tension-free closure of incisional wounds. It is also promoted as being able to integrate better than synthetic mesh into the healing matrix and resist to infection, essentially expanding its potential use in areas where the synthetic mesh is otherwise contraindicated [68] (EL 3). In accordance with the guidance set by the ventral\hernia working group, biological prosthesis should be implanted only in clinical cases where is present a contaminated or potentially contaminated surgical field as previous site infection, the presence of a stoma, accidental or programmed violation of the gastro-intestinal tract or a history of mesh infection or removing an infected mesh [69] (EL 3). Therefore, the attention on literature has been specifically focused on the biologic mesh in ventral hernia repair under contaminated conditions [70] (EL 2). To date, there have been four clinical reviews which concluded that biologic mesh should be incorporated in every surgeon’s armamentarium, especially in the setting of contaminated fields, but a closer look at the primary studies reported in these reviews indicates that such positive conclusions are not at all warranted, for several reasons. The primary literature that served as the basis for this conclusion consisted entirely of case series that varied widely in terms of sample size, mesh material used, how the mesh material was placed, and how the results were reported [68] (EL 3), [69] (EL 2), [71] (EL 3), [72] (EL 4) and another systematic review included biologic meshes as part of a larger discussion on available prosthetics for ventral hernia repair [73] (EL 3). This inconsistency makes it difficult, if not impossible to compare studies and drawing conclusions from them premature. However, biologic grafts offer the potential to perform single-staged repairs of contaminated or potentially contaminated ventral hernias with a real improvement in terms of social, economic and emotional costs if compared to lead towards a planned hernia, as in the past [73, 74] (EL 2–3). But, despite the rapid acceptance of these different materials into the surgical community, there are still many unanswered questions about biologics as their mechanism of action, ideal source material, the effect of post-harvesting processing methods and most appropriate placement techniques [75, 76] (EL 2).

Theoretically, they provide a scaffold for native cellular regeneration and neovascularization. Several animal experiments and case reports of re-exploration and biopsy evaluation have confirmed neovascularization and fibroblast deposition, but in not contaminated fields [77] (EL 2). The ability to rapidly vascularize, in a potentially infected field, might prevent bacterial colonization, degradation and graft failure. Recent animal models have confirmed the varied ability of different biologic grafts to clear bacterial contamination [78] (EL 3). Apart from the animal and tissue of origin, biological meshes differ in the de-cellularization and sterilization processes, material size and thickness, as well as the presence of cross-linking. Cross-links are bonds that link the collagen chains together and are present to a degree in all natural collagens. Additional cross-linking resists the degradation of collagen scaffolds by either host or bacterial collagenases and is claimed to enhance mesh durability. Moreover, in human beings, the biological response might be influenced by post-harvesting processing which modified the characteristic of the graft, as in the case of a cross-linking process which promotes a resistance to matrix metalloproteinase and stabilizes the structure of collagen, that lead to a long-lasting surgical implant [79] (EL 2). The theoretical advantages of cross-linked meshes may result in lower hernia recurrence rates, especially in complex hernia repair and contaminated/infected fields but the methodologic quality of the studies about this topic was generally poor, common reporting weaknesses included the following: lack of reporting wound classification, lack of a control group, failure to provide information on surgical technique and outcomes, failure to report which patients were lost to follow-up evaluation, and failure to provide biological mesh-specific information on hernia recurrence. Nevertheless, histologically, non-cross-linked materials exhibited greater cellular infiltration, extracellular matrix (ECM) deposition and neovascularization compared to cross-linked [80–83]. These results may explain the report of a relatively higher infection and explantation rates observed in cross-linked compared to non-cross-linked grafts [84] (EL 2). In contrast to these data a recent systematic review comparing cross linked and not cross-linked porcine meshes showed that more mesh infections occurred in the latter group. Findings appear to be controversial and at the light of these results, the surgeon has to face the choice of a graft to implant, balancing between mesh strength and durability or optimal tissue integration [76] (EL 2). Furthermore, the effectiveness of the biological mesh has subsequently been questioned in the case of surely infected fields, in which the incidence of surgical site occurrence (SSO) is similar to synthetic material use [85] (EL 3). Particularly the most frequently described major complication in biologic graft abdominal wall repair was recurrence ranging from 0 to 100%, with an overall weighted recurrence rate of 15.2% [86] (EL 3). These results could be widely influenced by appropriate graft placement techniques [87] (EL 2) and as showed, bridge repair without a midline restoration is affected by highest recurrences rates as 46.5% of cases, especially among non-cross-linked grafts [84] (EL 2). The level of contamination in the surgical site may also influence the recurrence rate by higher level of explanted meshes especially among cross-linked graft [88] (EL 2). The component separation, described for the first time by Ramirez [89], may address the problem, restoring anatomy and physiological function of the abdominal wall and by giving much more strength, through mesh interposition [90] (EL 1). As confirmed in a prospective multicenter randomized trial (RICH Study) [91] (EL 1) on a total of 80 patients with ventral incisional hernia repair in contaminated surgical field, the midline closure, with or without component separation and associated to a dermal porcine non-cross-linked graft, shows at 24 months after operation 25% recurrence rate in patients, with defects ranging from 203 to 220 ± 150 cm^2^, so close to the outcome following synthetic mesh repair in not contaminated surgical fields [92] (EL 1). Moreover, a proposal for a decisional model about the biological mesh use came from “Italian Biological Prosthesis Work-Group (IBPWG)”. According to this study, using a scoring model that combines infection grade with tissue lost grade, it would be is possible to achieve information about the best biological mesh choice [93] (EL 3).

From the limited studies that are present in the literature, there appears to be an acceptable overall recurrence rate of 20% and an infection rate of 24% [94] (EL 1). However, the evidence in the literature suggests that the total number of reported studies using biological mesh in infected fields for abdominal wall reconstruction in humans is low and most studies are of poor methodology.

7. When a synthetic mesh may or must be used in a complex abdominal wall repair?

All complex ventral hernia repairs (VHR) should be reinforced with prosthetic repair materials, but there is a significant increase in the risk of postoperative occurrences using mesh in clean-contaminated and contaminated cases compared to clean cases (WEAK RECOMMENDATION).

The use of monofilament and macroporous non-absorbable meshes, in extraperitoneal position, in the setting of the complex abdomen with contamination seems a cost effective approach (WEAK RECOMMENDATION).

Fascial traction technique with synthetic meshes combined to NPWT therapy in temporary abdominal closure shows a lower rate of enteroatmospheric fistula respect the use of mesh alone (synthetic and biologic) in inlay position (WEAK RECOMMENDATION).

Further clinical trials are needed to characterize the use of new synthetic bioabsorbable 3D and 2D meshes in complex abdominal wall repair.

All complex ventral hernia repairs (VHR) should be reinforced with prosthetic repair materials [95, 96] (EL 2), [97] (EL 3), but there is a significant increase in risk of postoperative occurrences using synthetic mesh in clean-contaminated and contaminated cases relative to clean cases [69] (EL 3), [98, 99] (EL 2), [100] (EL 3). The use of monofilament and macroporous non-absorbable meshes in the setting of a complex hernia and/or contamination seems, in extraperitoneal position, a cost-effective approach than bioprosthetic mesh [101] (EL 2), [102–109] (EL 3). Fascial traction technique with synthetic meshes combined with VAC therapy in temporary abdominal closure shows a lower rate of enteroatmospheric fistula respect the use of mesh alone (synthetic and biologic) in inlay position [45] (EL 2), [110] (EL 5). Early results of a multicentric and prospective study on VHR using synthetic bioabsorbable 3D mesh demonstrate that this mesh in contaminated fields, in intra and extraperitoneal position, shows the ability to resist infection and remodel by the time with good results in terms of infection and recurrences rate [111] (EL 4), [112–114], (EL 3). In literature are present only pre-clinical studies and case reports, consequently, further clinical studies are needed to better characterize the use of new synthetic bioabsorbable 2D meshes in complex abdominal ventral hernia repair [115–118] (EL 4).

8. Biological prosthesis implant in pediatric and neonatal surgery.

The use of biological prosthesis is safe and feasible in pediatric abdominal wall closure. The use of the biological prosthesis in a contaminated surgical field improves the surgical outcome without the need of prosthesis removal in case of infection. The use of biological prosthesis allows the abdominal wall closure after pediatric abdominal transplantation. The use of biological prosthesis allows the abdominal wall closure in patients with congenital abdominal wall defects (WEAK RECOMMENDATION).

Repair of congenital abdominal wall defects, diaphragmatic hernia with abdominal muscle aplasia, and abdominal wall closure of pediatric transplant recipient with donor size discrepancy have seen the use of prosthetic patches of non-absorbable materials which represented a valid solution but can be a source of infection and complications. Biologic grafts have been introduced as an alternative to synthetic mesh.

No systematic reviews were found for the pediatric population.

There are few and limited studies published on the application of biologic mesh for pediatric abdominal wall closure. They are very heterogeneous, mainly because they describe different kinds of graft, different patient characteristics and pathologies, different surgical indications and techniques. Then a comparison of the data in the literature is really difficult.

The two more recent reviews conclude that biologic grafts perform similarly to synthetic mesh for incisional hernia repair and they are associated with a high salvage rate when infected [86, 88] (EL 3).

Other two reviews suggest that cross-linked mesh has the best clinical outcomes in contaminated or infected fields [119, 120] (EL 3).

The last review affirms that allograft acellular dermal matrix have a higher recurrence rate as compared with xenograft products [121] (EL 3).

Regarding the use of the biological prosthesis in congenital abdominal wall defect, only one study was identified. It is about repair of a congenital diaphragmatic hernia. 15/118 patients were treated with an abdominal patch (eight with diaphragmatic patch, seven with primary diaphragmatic repair). In this group, the mortality rate was significantly higher. A similar trend was also observed in diaphragmatic patch versus non-patch group [122] (EL 4).

Other studies suggest that biological mesh (Permacol, Surgisis, Strattice) allow complete abdominal closure after transplant (liver, intestine, kidney, multi-visceral) in children with donor size discrepancy. Biological prosthesis seems to have long-term durability with no incisional hernia on short and medium-term follow-up [123–127] (EL 4). The three articles selected assert that Permacol was effective for the reconstruction of the abdominal wall defect in particular cases: multi-trauma, conjoined twin, and in assisting abdominal wall closure of pediatric renal transplant recipient [124, 128, 129] (EL 4).

In pediatric patients affected by giant omphalocele, the biologic mesh was applied as a primary abdominal fascia substitute with good results, no fascial dehiscence or infection [130–132] (EL 4). Surgisis was implanted for abdominal wall defects due to different pathologies: omphalocele, gastroschisis, ventral hernia after diaphragmatic hernia repair, omphalopagus conjoined twins and liver transplantation. In these case series, some complications occurred: wound infection, seroma formation, and recurrence. None required patch removal [125, 126, 133–135] (EL 4).

The low level of evidence of the case studies presented, the lack of randomized studies, the lack of age-related pediatric review only allow us to suppose that there is a good outcome with the use of biological prostheses. We cannot then formulate which are the real clinical indications for the use of a biological prosthesis rather than a synthetic one. This is the same conclusion shared by the latest reviews about adult population. Randomized controlled trials are necessary to determine the right application of biologic prosthesis.

9. Which follow-up in the biological mesh?

Patients with biological prosthetic repair of the complex abdomen should undergo careful follow-up with outpatient clinical examination every 12 months after surgery up to 5 years of follow-up. Suspected recurrent hernia or wound complaints should be evaluated by abdominal CT or MRI study (STRONG RECOMMENDATION).

Three biological grafts were found almost exclusively in the Literature in ventral incisional hernia repair (VIHR) in critical conditions and were included in this analysis: Alloderm^®^, Permacol^®^, Strattice^®^ and Surgisis^®^.

A recent review [74] (EL 3) on studies with at least Lev 4 evidence and at least 40 patients undergoing VIHR came up with 461 total patients. Contamination (Grade 4, according to the grading system of ventral incisional hernia introduced in 2010) or clean/contamination (grade 3) was present in 32–100% of the cases, depending on the study and the type of prosthetics used. The mean follow-up for Alloderm^®^, Strattice^®^ and Surgisis^®^ patients was 25.9, 24 and 20.5 months, respectively and recurrence rates 30.1, 28 and 18.1%. With an average follow-up of only 25 months in the whole cohort of patients, it was not easy to draw conclusions regarding long-term results. Such short follow-up is concerning, considering that most recurrences could take place outside the 2-year follow-up window. We don’t know if there is any ‘plateau’—at some point—in the recurrence rate. In Itani’s paper [91] (EL 4), nearly half of the infection-related complications (including abscesses and sinuses) described after using Strattice^®^ in dirty, contaminated and clear/contaminated cases were late-occurring. Hernia recurrences were 19% by month 12 and 28% by month 24. Therefore, both the temporal relationship between repair and recurrence and repair and occurrence of complications, which is different for each mesh and is missing in the literature, would be of great interest. This could be available only with a strict follow-up and a meticulous retrieve of data from outpatient clinical examination. A ‘freedom from recurrence’ point could be defined only after scrutiny of long-term follow-up of prospective studies and also a precise definition of postoperative complications might require prolonged follow-up. As reported in Jin study on Alloderm^®^ [136] (EL 4) their median patients’ follow-up was 21.4 months (range 15–36 months) and the mean time to hernia recurrence was 1 year, although recurrences occurred at 31 months, indicating the importance of continued follow-up.

An interesting review [137] (EL 3) focused on the claimed superiority of biological over synthetic mesh in VIHR under contaminated conditions. Four clinical reviews and one systematic review were identified with a total of 25 primary articles: only ten had a mean follow-up time longer than 12 months and yet they all concluded supporting the continued use of biological meshes in contaminated fields. To note, quite a few studies reported only perioperative outcomes and in studies, with longer follow-up a range of follow-up was given but the total number of patients available for follow-up was missing. In this review the follow-up ranges for Alloderm, Surgisis and Permacol were, respectively: 6–22, 14–29 and 11–18 months. The short-term picture provided by most articles on biological mesh use in VIHR makes a comparison with synthetic mesh quite complicated, because of different follow-up times. Recurrence occurring after the short-term period of follow-up might be underestimated. In this light, the early termination of the LAPSIS trial (randomized study of Surgisis^®^ vs. classical synthetic mesh for clean primary ventral and incisional hernia) should be kept in mind: the higher preliminary recurrence rate in the biological mesh group compared with the synthetic one was one of the key factors for the premature interruption of the study [138] (EL 2).

Alloderm^®^ (based on the acellular human dermal matrix) represents an example of the importance of long-term follow-up to better define the characteristics and the temporal behavior of different meshes. It is the biological mesh with longer follow-up available in the Literature. It has been reported to “relax” over time: high elastin/collagen ratio, insufficient pre-stretching, lack of additional cross-linking, and thin and vulnerable border regions because of the dermatome harvest from human cadavers might be responsible for bulging and eventration [88] (EL 2). Actually, laxity does not necessarily affect patient’s functionality and is not clear, yet, if and how it affects patients’ life. So, longer follow-up studies on quality of life data might help to solve this issue. The idea that chemical cross-linking affects and significantly reduces the overall stretch of the elastin fiber found in all dermis-based grafts is a hypothesis and as such needs to be tested in the long-term follow-up to confirm better preliminary short-term results of cross-linked porcine dermis derived meshes compared to Alloderm^®^. On the other side, if it is true—as suggested by recent reports [139–141] (EL 3–4) that long-term graft stretching may occur even after pre-stretching of the graft, then the diastasis and/or hernia recurrence rates can be assumed to increase over time as the graft continues to stretch and weaken.

The ongoing SIMBIOSE randomized multicenter controlled study, comparing the use of mesh versus standard wound care in infected incisional ventral hernia will have preliminary data available after 6 months, but is correctly planned to last till a 3-year period of follow-up is reached, before correct definitive conclusions on use of biological mesh in contaminated field can be drawn [142] (EL 3).

An interesting report [143] (EL 4) was recently published on single-staged repair of infected and contaminated abdominal wall defects with biological implants on 128 patients (including 102 Strattice^®^ and 16 Alloderm^®^). With a mean follow-up of 21.7 months (range 1–73.8 months) and an overall recurrence rate of 31.3%, this paper revealed that hernia recurrence truly presented as a broad-spectrum temporal event: hernia recurrences occurred as early as 5.6 months and as late as 73.4 months after surgery and the mean time to recurrence was over 2 years. Authors of this study called into question whether biological repairs should be—realistically—considered ‘temporary’ reinforcement barriers, acting as a mean of delaying inevitable recurrence of a hernia in complex fields.

A recent review [120] (EL 3) compared recurrence rates obtained by different biological meshes, suggesting Permacol^®^ had the lowest failure rate and the longest time to failure, particularly in contaminated fields. The authors of this review included Shaikh study on 20 patients with acute and chronic abdominal wall defects and meshes placed in potentially contaminated fields (15% recurrence at 18-month follow-up), Catena study on seven patients with incisional hernias in contaminated fields (no recurrence at 11.2 months) and Parker study including also five patients with contaminated wounds (class II–II–IV) and one recurrence at 13 months of follow-up.

Studies with longer follow-up are awaited to assess the durability over time of biological prosthetics, given their biodegradable nature. Although biological meshes represent one of the most promising alternatives to a two-stage repair in compromised abdominal fields, still a more convincing evidence of their long-term results is necessary to overcome resistance to their use, especially in Europe, due to their high cost.

10. Complications and their treatment after biological mesh implant.

Infectious events are the most relevant complications. Controversial findings of surgical site infection rate do not allow identifying clear differences between cross-linked and not cross-linked mesh. The surgical site occurrences are usually managed non-operatively or require minimal operative treatment. Mesh removal is uncommon (WEAK RECOMMENDATION).

Surgical complications after abdominal wall repair with biological mesh are mainly related to wound events and they can define as surgical site occurrence (SSO). A recent systematic review, including 1.152 patients, documented an SSO rate of 46.3% [88] (EL 2). The comparison of morbidity between wound classes regarding infection and total surgical morbidity showed that both were dependent on wound class [88] (EL 2). The infection and total surgical morbidity were significantly higher in the contaminated/dirty group than in the clean/clean-contaminated group [88] (EL 2). The SSO according to the ventral hernia working group’s (VHWG) grading system could not be evaluated accurately because of inadequate study details of several reports [86] (EL 2). However, an author found a lower rate of complications in grade 3 patients, when biologic is compared with synthetic mesh [144] (EL 3). A recent metanalysis reported no significant difference in wound complication rates in ventral hernia repair with human-derived biologic grafts compared with porcine biologic mesh [145] (EL 2) but data in the literature are still controversial. Another systematic review, including 60 studies with a total of 1.241 patients, reported an SSO rate of 52.8%, and combining mesh product by a source the rate was: human dermis 48.3%, porcine small intestinal submucosa 82.6%, xenogenic dermis 50.7%, xenogenic pericardium 6.3% [86] (EL 2). Additionally, another Author analyzed SSO divided by biologic graft with regard to infection, seroma formation and total surgical morbidity and human dermis and porcine intestine had a higher rate of infectious than porcine dermis, as well seroma formation rate: total surgical morbidity was lower with porcine dermis comparing with human dermis and porcine intestine [88] (EL 2). Furthermore analyzing and comparing data of all porcine cross-linked mesh with porcine non-cross-linked, it was shown that infection rates were 9.1% for porcine cross-linked and 18% for non-cross-linked [86] (EL 2). Comorbidities such as hypertension, diabetes mellitus coronary artery disease, ASA score, are not in correlation with wound complications, while age > 60 years, BMI, long surgical time, use of large or more than one sheet of prothesis were associated with an SSO [146–148] (EL 2). Surgical site occurrence was divided into infectious and non-infectious complications. Infectious complications include purulence, cellulitis, positive culture, chronic wound infection, abscess, enterocutaneous fistula and mesh infectious, whereas edema, seroma, hematoma, wound dehiscence, skin necrosis, bleeding and fluid collections were considered non-infectious [86, 145] (EL 2–3). In a metanalysis comparing biologic versus non-biologic mesh, the former demonstrated a lower rate of infectious wound complications and an equivalent rate of non-infectious wound complications when compared with non-biologic mesh [86] (EL 3). In the literature, infection rates range from 5.4 to 57.1% when a biologic mesh is used for ventral hernia repair, however, the wound complication rate of 10.9% in this metanalysis was lower than the 36.5% of the mean complication rate for synthetic mesh [86] (EL 3). A systematic review reported a wound infectious rate of 15.9%, but the majority of infections were superficial [88] (EL 2) and other infectious complications were intrabdominal abscesses in 2.4% and miscellaneous in 2.7%. The postoperative course was complicated by an enterocutaneous fistula in 6.5% of cases, of this 56% was related to fistula takedown performed concomitantly with the hernia repair, 10.4% after bowel surgery and 12.6% in patients with open wounds or after simple ventral hernia repair [88] (EL 2). A prospective controlled study (RICH study) revealed as in 67% of patients a minimal treatment was required to treat the postoperative infections [91] (EL 1). They can manage successfully by incision and drainage with antibiotic therapy. In front of this, a majority of the patients were able to maintain the reconstruction without the need for explantation [91]. Systematic reviews confirm the need for removing the grafts in only 4.9% and 2% of patients [86, 88] (EL 2–3). Whereas after synthetic mesh repair, mesh removal is mandatory when infection develops. A retrospective cohort study showed the hernia grade as the only independent factor associated with mesh explantation [149] (EL 2). Patients with a hernia grade of 4 had 15-fold increased risk of mesh explantation when compared to patients with hernia grade 1 and the rate of mesh explantation for grade 1 and 2 hernias were similar between biologic and synthetic mesh [149] (EL 2).

Related to non-infectious complications, an author reported a seroma formation of 14.2% [88], of these five cases of explantation, were documented after significant seroma formation in a report of repair with porcine intestine mesh [150]. Haematomas incidence rate was reported 3% on 354 patients [88] (EL 2). In another review, seroma/hematoma was the second most common complication reported and occurred in 12% of patients [86] (EL 2). Most seroma resolved either spontaneously or after percutaneous aspiration requiring a bedside or outpatient intervention (21.4% of patients) and in few cases (14.2%) a surgical intervention [86] (EL 2). Other postoperative wound-related complications were skin necrosis/breakdown (16.9%) and graft rejection/degradation (2.5%) in a series, while another reported superficial dehiscence 3.8%, skin necrosis 0.9%, mesh disintegration 0.5% and flap necrosis 0.3% [86, 88] (EL 2). Deep wound dehiscence necessitating operative intervention was documented in 8.6% of 191 patients [88] (EL 2). In cases of skin necrosis involving deep tissue, the mesh can be exposed. Operative debridement of the wound is necessary to determine the extent of necrosis and choosing the right dressing will ensure the hydration to the biologic graft. Option for dressing includes hydrating gels, enzymatic debridement agents, micro-debridement dressing and VAC therapy [151] (EL 4). Mortality after ventral hernia repair with biologic mesh is usually unrelated to biologic prothesis (e.g., multiple organ failure, congestive heart failure, and disseminated intravascular coagulation). Systematic reviews reported an overall mortality rate of about 4% and a 30 days mortality of 2.3% [86, 88] (EL 2).

11. The use of negative pressure wound therapy (NPWT) in complex abdominal wall repair’s SSO (Surgical Site Occurrence) with synthetic meshes and biological implants.

NPWT can be safely used both with synthetic and biologic infected meshes (WEAK RECOMMENDATION).

NPWT is useful because promotes granulation tissue formation and tissue ingrowth over the synthetic mesh (STRONG RECOMMENDATION).

NPWT could reduce hospital-stay with a decreased rate of complications (WEAK RECOMMENDATION).

Negative pressure wound therapy (NPWT) has shown many promising results in different types of wounds. The evidence for the effects of NPWT in reducing SSI and wound dehiscence remains unclear, as does the effect of NPWT on time to complete healing [46]. The use of NPWT in the case of an open abdomen or split-thickness skin graft was well established in practice and the clinical guidelines have been readily available [152, 153]. Several therapeutic strategies have been advocated in the past to treat deep mesh infections, ranging from complete excision of the mesh, local excision of some parts of the mesh to local drainage with concomitant intravenous antibiotics [154–156]. A possible alternative to the above methods is the NPWT, which is a non-invasive and dynamic way to obtain mesh coverage and adequate wound closure [157–159] (EL 2). NPWT can be used in the treatment and salvage of infected meshes after hernia repair [159–161] (EL 2). Best results can be achieved in the case of infection of deep mesh with a large pore size (2–5 mm) when compared to small pore size meshes. The wider pore size favors tissue ingrowth, leading to better incorporation and diminishing the mesh surface area [162]. The difference between polyester and polypropylene is still under debate [163–165]. The affinity of different meshes to bacteria has also been studied, and it is established that microporous and multifilament meshes do have a higher affinity for bacterial contamination [165, 166] (EL 2).

Biologic mesh performs like a scaffold for the granulation tissue that is stimulated by the negative pressure. The mesh must be covered by Vaseline gauze or polyvinyl alcohol foam (interface dress) to prevent adherence with the implant. NPWT should start at − 75 mmHg pressure, then progressively increase at − 125 mmHg continue pressure. Sometimes the implant can break or flake and early granulation can be observed inside the mesh perforation [166–168]. NPWT promotes granulation tissue formation through the mesh, that occurs 1–7 weeks after its implant [158] (EL 2).The formation of granulation tissue allows the application of a skin graft on the biologic material [167] (EL 4).

Future multi-site, prospective, controlled studies would provide a strong evidence base from which treatment decisions could be made in the management of these challenging and costly cases. Additionally, cost–benefit studies examining the use of newer adjunctive technologies, such as V.A.C. Instill^®^ Wound Therapy and GranuFoam Silver (KCI Licensing, Inc., San Antonio, TX) dressing, earlier in the treatment course, are needed in addition to the use of alternative biologic meshes of both human and porcine matrices [158, 159, 166, 167] (EL 3–4).

12. The recurrence after biological mesh implant.

Hernia recurrence after abdominal wall repair with biological mesh is comparable to repair with non-biologic mesh. No significative differences in recurrence rate are found among several types of biological meshes (WEAK RECOMMENDATION).

There is an evident correlation between hernia recurrence and postoperative infection as well as repair performed in contaminated field and in high-risk patients. The use of biological mesh in bridge technique results in a high recurrence rate, thus the fascial closure with or without component separation technique should be achieved when possible (STRONG RECOMMENDATION).

The prosthetic mesh can be used safely in recurrent hernias, without biological mesh removal (WEAK RECOMMENDATION).

The most frequently described major complication after ventral hernia repair with biological mesh is recurrence. The reported frequency of recurrence varied from 0 to 100% with an overall weighted recurrence rate of 15.2% [86] (EL 3). Hernia recurrence is defined as any kind of repair failure, including postoperative hernia, laxity, bulge, eventration, or diastasis [145] (EL 2) and is based on clinical examination findings; only those patients with suspected hernia recurrence usually undergo confirmatory imaging studies and probably if all patients were imaged, the incidence of recurrence would likely be higher [169]. In assessing recurrence rates, the duration of follow-up is a key issue for two reasons: short-term follow-up can underestimate the recurrence rate and the results of two studies cannot be compared unless the follow-up duration is similar. Short term recurrence are likely to be due to either technical issue, as mechanical failures perhaps resulting from an inequality between high tension and force placed on the mesh, and very high-risk patients (smoking, obesity, pulmonary disease); whereas late recurrences suffered from prolonged wound healing and infectious complications [120, 169] (EL 2–3). Thus, recurrence rate increase among the length of follow-up, as in a prospective study with non-cross-linked porcine dermal mesh the recurrence rate is 19% at 12 months and 28% at 24 months [91] (EL 1). A longer follow-up, like 5 years, would answer if the biological mesh is reliable as definitive repair in high-risk patients [170] (EL 3). In a metanalysis, the average recurrence rate for ventral hernia repair across all the studies was 17.1% and was not significantly different between biologic mesh (18.6%) and non-biologic mesh (15.7%) [145] (EL 2) and there was no significative difference in recurrence rates for human-derived and porcine-derived biologic grafts [145] (EL 2). The risk factors influencing recurrence are patient factors (increased intra-abdominal pressure, diminished tissue integrity) and technical factors (infection, lateral mesh distraction, missed hernia) [69] (El 3). It is estimated that more than 75% of all recurrence is due to infection and inadequate repair material fixation and/or overlap [69] (EL 3). However, wound infection appears to significantly increase the risk for hernia recurrence [69] (EL 3) [169] (EL 2). Furthermore, a systematic review found that across all studies, hernia recurrence correlated with the presence of postoperative infection [88] (EL 2). There were significantly fewer recurrences in clean fields compared with contaminated or infected fields and the complex/high-risk patients, even at short term follow-up [88, 120] (EL 2). Whereas one study reported an overall recurrence rate of 30% in contaminated fields [171] (EL 3), some Authors found a strong correlation between the wound class and the recurrence rate, with a 39% rate in dirty fields against 4.5% in clean fields [172] (EL 3). Whether mesh should be used as an onlay, sublay or bridge prosthesis remains a source of debate within the surgical literature. In a metanalysis biologic mesh combined with component separation technique (CST) reported a recurrence rate of 18.5% that is higher than the 11.4% recurrence rate of CST with non-biologic mesh [145] (EL 1). If the fascial closure, with or without CST, is achieved the recurrence rate is lower than no fascial closure, as in bridge technique, 23% vs 44% in 24 months of follow-up [91] (EL 1). The largest published experience with human dermal mesh used as bridge demonstrates recurrence in 20.2% of cases [173] (EL 3). A retrospective study on porcine dermal cross-linked mesh reveals a recurrence rate at 5 years follow-up, with reinforcement technique by onlay or sublay placement, of 20 and 53%, respectively, and an 80% recurrence when bridge technique was used [170] (EL 3). Some authors report problems with bulging and diastasis at the repair site when human dermis is used, probably due to stretching of the mesh [140, 174] (EL 3). In a retrospective study comparing bridged and reinforced technique the only predictive factor for hernia recurrence was the use of the bridging technique [136] (EL 3). When recurrence occurs, it can be successfully repaired with prosthetic mesh even in minimally or potentially contaminated field without explantation of previously biological mesh [175] (EL 4).

13. Nutritional and antimicrobial support treatment.

Early enteral nutrition in the open abdomen should be considered in all patients with a viable gastrointestinal tract (STRONG RECOMMENDATION).

An antibiotic perioperative prophylaxis is essential in all cases. In the case of gross contamination, a therapy of broad-spectrum antibiotics should be continued for 4–7 days or more according to clinical conditions (WEAK RECOMMENDATION).

The advantage of early Enteral Nutrition (EN), like reduction in septic complications, in the patient with a severe injury, is well documented. Its application in those patients with an open abdomen has yet to be defined. One study reports increased fascial closure rates with the initiation of EN before day 4 after injury [176] (EL 3) Another study suggests a reduced incidence of ventilator-associated pneumonia with early EN but shows no impact of EN on abdominal closure rates [177] (EL 3). In Patients with an intact gastrointestinal tract, Moore et al. have shown it is safe to feed the patient with an open abdomen. These benefits appear to decrease the time for abdominal fascial closure, decrease complication rates, decreased mortality. Although EN for patients with bowel injuries does not seem to alter fascial closure rates, complications, or mortality [178] (EL 3). The open abdomen does not warrant any additional period of antibiotic coverage. In most cases, perioperative antibiotic treatment is all that is required [179] (EL 3). There is no difference in the complication rate between prophylactic and prolonged antibiotic use [180] (EL 3). For patients with complicated intra-abdominal infection, no unique antibiotic strategies are recommended. Therapy should be limited to 4–7 days, as was recommended in the Surgical Infection Society guidelines for complicated intra-abdominal infections [181] (EL 2).

## Discussion

Although in elective surgery the term “complex abdomen” is often used as a synonym for “complex abdominal wall hernia”, where the main problem is to face a technically challenging and time-consuming hernias, in emergency situations to close and how the abdominal wall might represent, in particular and demanding situations, a surgical nightmare to solve by surgeons. As it is intuitive and despite the problem’s frequency, there is no an easy and unique solution for a complex abdomen. The treatment as to be often tailored and individualized, bringing in mind two priorities.

The first one is to control or, if possible, to prevent “the causative source” and its related complications. In the first situation, decompressive laparostomy should be considered in a case of abdominal compartment syndrome in patients with critical conditions or after the failure of a medical treatment or less invasive methods. In the second one, beyond different mechanism, patients with surgical emergency diseases might reach the same pathophysiological end point of trauma patients where a preventive “open abdomen” might be indicated. A temporary abdominal closure might be achieved with different techniques: in the case of a non-infected field, the Wittmann patch, and the NPWT had the best outcome followed by meshes (to prevent also the lateral fascial retraction). In the case of an infected field, NPWT techniques seem to be the preferred in both the situations, the main goals to be achieved are the definitive closure, as soon as possible, of the abdomen. Within 9 days has been reported to be the ideal time.

The second priority is to create optimal both general as local conditions for healing: the right antimicrobial management, feeding—preferably by the enteral route, and managing correctly the open abdomen wall. No doubt that to minimize hernia recurrences, the use of a mesh appears to be—if and when possible—the gold standard. There is a lot of enthusiasm about biological meshes. But the actual evidence supports their use only in contaminated or potentially contaminated fields but above all, to reduce the higher rate of recurrences, the wall anatomy and function should be restored in the midline, with or without component separation technique. On the other site has not to be neglected that the use of monofilament and macroporous non-absorbable meshes, in extraperitoneal position, in the setting of the complex abdomen with contamination, seems to have a cost effective role too.

The treatment of a patient with a complex abdomen requires not only a deep understanding of the treatment options and consciousness of all the possible morbidities but the involvement of different specialties, keeping in mind that the key issues are timing and coordination.

The idea of this consensus conference was mainly to try to bring order in the so copious, but not always so “evident” literature utilizing and exchanging the expertise of different specialists.
